# Impact of COVID-19 Vaccination on Mortality and Clinical Outcomes in Hemodialysis Patients

**DOI:** 10.3390/vaccines12070799

**Published:** 2024-07-19

**Authors:** Rihong Hu, Jiazhen Yin, Tingfei He, Yuxuan Zhu, Ye Li, Jinchi Gao, Xiaomin Ye, Lidan Hu, Yayu Li

**Affiliations:** 1 Key Laboratory of Kidney Disease Prevention and Control Technology, Department of Nephrology, Hangzhou TCM Hospital of Zhejiang Chinese Medical University (Hangzhou Hospital of Traditional Chinese Medicine), 453 Tiyu Road, Xihu District, Hangzhou 310012, China; hzhurihong@163.com (R.H.); yexiaomin20096844@163.com (X.Y.); 2 Hemodialysis Unit, Hangzhou Hospital of Traditional Chinese Medicine, 453 Tiyu Road, Xihu District, Hangzhou 310012, China; 3 Hangzhou Clinical College, Zhejiang Chinese Medical University, 548 Binwen Road, Hangzhou 310053, China; yinjiazhen@163.com (J.Y.); s1137072939@163.com (T.H.); zhuyuxuan98@126.com (Y.Z.); taeyeon30935@gmail.com (Y.L.); gjc19857169066@163.com (J.G.); 4 Department of Nephrology, The Children’s Hospital, Zhejiang University School of Medicine, National Clinical Research Center for Child Health, 3333 Binsheng Road, Binjiang District, Hangzhou 310003, China

**Keywords:** hemodialysis, COVID-19 vaccination, mortality, clinical outcomes, survival analysis

## Abstract

This study analyzed 550 hemodialysis patients, 469 unvaccinated and 81 vaccinated against COVID-19, to assess the impact on infection rates, mortality, and clinical/laboratory parameters. Gender distribution was similar (*p* = 0.209), but the vaccinated group’s median age was significantly lower (*p* = 0.005). Hospitalization rates showed no significant difference (*p* = 0.987), while mortality was lower in the vaccinated group (*p* = 0.041). Only uric acid levels were significantly higher in the vaccinated group (*p* = 0.009); other parameters, including creatinine and B-type natriuretic peptide, showed no significant differences. Age was an independent predictor of mortality (HR = 1.07, *p* < 0.001). Peak mortality occurred in December 2022 and January 2023, predominantly among unvaccinated patients. Although vaccination lowered mortality, it did not significantly affect long-term survival rates (*p* = 0.308). Logistic regression identified age and dialysis duration as significant mortality factors. Monthly death counts indicated higher mortality among unvaccinated patients during peak pandemic months, suggesting that vaccination provides some protection, though no significant long-term survival benefit was found.

## 1. Introduction

COVID-19 has a significant impact on hemodialysis patients, as chronic kidney disease is considered a key independent risk factor for COVID-19 mortality [1]. Epidemiological studies indicate that hemodialysis patients have a markedly increased susceptibility to COVID-19 [2,3]. Research shows that hemodialysis patients who are infected with COVID-19 are more likely to experience severe outcomes [4]. The risk of COVID-19 infection is particularly high among patients undergoing in-center dialysis, categorizing them as a high-risk group [5]. The inherent congenital and adaptive immune cell dysfunctions in hemodialysis patients [6], combined with frequent hospital visits, dialysis in enclosed spaces, and social interactions during hemodialysis sessions [7], significantly contribute to their increased susceptibility. Additionally, kidney disease exacerbates the risk of in-hospital mortality for COVID-19 patients [8]. Studies also emphasize the elevated infection risk due to comorbid conditions and the necessity for frequent visits to dialysis centers [9]. It is recommended that routine COVID-19 screening for hemodialysis patients be implemented to facilitate the early detection of asymptomatic cases and prevent outbreaks within dialysis units. These studies underscore the critical importance of COVID-19 vaccination for the hemodialysis population [10].

Recent years have seen extensive research into the efficacy of SARS-CoV-2 vaccines among the hemodialysis population. Studies have shown that vaccination significantly reduces the rates of COVID-19 infection, hospitalization, and mortality among hemodialysis patients [11,12,13,14]. Current research indicates that, under the dominance of the Omicron variant, the efficacy of the CoronaVac vaccine is relatively weaker compared with mRNA vaccines [15,16,17]. However, in China, the majority of hemodialysis patients have been vaccinated with the traditional inactivated virus vaccine, namely CoronaVac. This study focused on the effects of the CoronaVac vaccine, aligning with China’s primary vaccination strategy. Our research aimed to evaluate the relationship between vaccination and hospitalization and mortality rates, explore the correlation between certain laboratory indicators and COVID-19 vaccination over a two-year period, and assess the protective effects and long-term efficacy of the CoronaVac vaccine in Chinese hemodialysis patients. This approach allowed us to compare groups receiving different vaccine doses and provided a clearer depiction of the situation specific to the Chinese context.

## 2. Materials and Methods

### 2.1. Study Population

This study included patients undergoing chronic maintenance hemodialysis at the Hemodialysis Unit of Guangxing Hospital, affiliated with Zhejiang Chinese Medical University, in November 2022. Patients were screened based on specific exclusion criteria to ensure the homogeneity of the study population. This study was conducted in accordance with the Declaration of Helsinki and received approval from the Ethics Committee of Hangzhou Traditional Chinese Medicine Hospital (approval number 2023KLL027). Written informed consent was obtained from all participants prior to their inclusion in this study.

*Exclusion Criteria*

Patients meeting any of the following criteria were excluded from this study: transferred to local or other hospitals (*n* = 438), transitioned to peritoneal dialysis (*n* = 22), underwent renal transplant (*n* = 13), experienced renal function recovery (*n* = 20), died before the COVID-19 pandemic (*n* = 3), or lost to follow-up (*n* = 37).

*Participant Selection*

**Figure 1:**
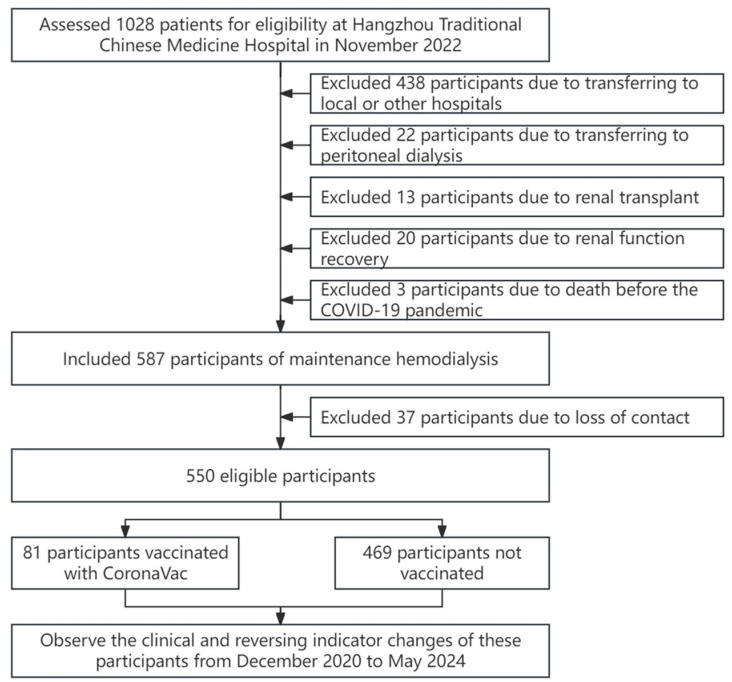
Flowchart of the enrollment process.

After applying the exclusion criteria, 587 patients undergoing maintenance hemodialysis were initially included in this study. Following the exclusion of 37 patients due to loss of contact, 550 eligible participants remained. These participants were divided into two groups: patients vaccinated with CoronaVac (*n* = 81) and unvaccinated patients (*n* = 469). See Figure 1 for details.

### 2.2. Data Collection

Data were sourced from the Hospital Information System (HIS) of the Hemodialysis Center at Guangxing Hospital affiliated with Zhejiang Chinese Medical University (Hangzhou Kidney Disease Hospital). We recorded the vaccination status of all patients undergoing maintenance hemodialysis, including primary immunization, booster doses, and secondary booster doses. Demographic and clinical data were extracted from registered patient medical records, including age, gender, and chronic comorbidities. We monitored symptomatic COVID-19 infections, COVID-19-related hospitalizations, COVID-19-related deaths, and overall mortality. Clinical parameters, including blood biochemical markers, hemoglobin levels, and dialysis-related metrics, were also monitored. These parameters were observed from December 2022 to May 2024 to capture any changes over time and assess the long-term effects of vaccination.

### 2.3. Statistical Methods

This study utilized various statistical methods to analyze the demographic and clinical characteristics (Supplementary Table S1), as well as survival outcomes, of 550 hemodialysis patients divided into vaccinated and unvaccinated groups. All analyses were conducted using SPSS (Statistical Package for the Social Sciences) version 26.0.

Chi-square tests were employed to compare categorical variables, such as gender distribution and hospitalization rates, between the vaccinated and unvaccinated groups. The Mann–Whitney U test, a non-parametric test, was used to compare non-normally distributed numerical values between the groups. Independent samples t-tests were applied to compare continuous laboratory parameters between the two groups. Kaplan–Meier survival analysis was performed to estimate the survival probabilities over time for the vaccinated and unvaccinated patients, with the log-rank test used to assess the statistical significance of differences between the survival curves. Cox proportional hazards regression analysis was conducted for both univariate and multivariate analyses to evaluate the impact of various clinical variables on survival rates. Univariate analysis identified significant predictors of survival, while multivariate analysis adjusted for multiple factors to determine independent predictors. Statistical significance was considered at *p* < 0.05.

## 3. Results

### 3.1. Demographic and Clinical Characteristics

This study analyzed 550 hemodialysis patients, with 469 unvaccinated and 81 vaccinated against COVID-19. Demographic and clinical characteristics were compared between these groups. No significant difference was observed in gender distribution (*p* = 0.209). The median age of the vaccinated group was significantly lower (*p* = 0.005). Hospitalization rates showed no significant difference (*p* = 0.987). Mortality rates were significantly lower in the vaccinated group (*p* = 0.041). Among clinical and laboratory parameters, only uric acid (UA) levels were significantly higher in the vaccinated group (*p* = 0.009). Other parameters, including creatinine (Cr), B-type natriuretic peptide (BNP), Kt/V, low-density lipoprotein cholesterol (LDL-C), parathyroid hormone (PTH), phosphate (PHOS), prothrombin time (PT), hemoglobin (Hb), albumin (ALB), total cholesterol (TCH), aspartate aminotransferase (AST), calcium (Ca), sodium (Na), β2-microglobulin (β2-MG), and ferritin, showed no significant differences between the groups. For detailed results, see Table 1.

### 3.2. Survival Analysis by Vaccination Status

The demographic and clinical characteristics of 550 hemodialysis patients were analyzed to evaluate the impact of COVID-19 vaccination on infection rates, mortality, and various laboratory indicators (Table 2). Patients were categorized into the Survivor Group (*n* = 477) and the Deceased Group (*n* = 73). No significant difference in gender distribution was observed between the groups (*p* = 0.810). The median age was significantly higher in the Deceased Group (77.00 years) compared with the Survivor Group (63.00 years) (*p* < 0.001). A greater proportion of patients in the Deceased Group required hospitalization (29.17% vs. 12.58%, *p* < 0.001). The incidence of severe COVID-19 was markedly higher in the Deceased Group (20.55% vs. 1.05%, *p* < 0.001). Vaccination status showed significant differences, with more vaccinated individuals in the Survivor Group (15.93% vs. 6.85%, *p* = 0.041), although the number of vaccine doses did not differ significantly (*p* = 0.264). In the Deceased Group, levels of BNP, UA, Cr, PTH, and AST were significantly increased, while levels of ALB and P were significantly decreased (all *p* < 0.05). No significant differences were found in Kt/V, LDL-C, PTH, Ca, Na, TCH, and ferritin levels. These findings highlight that age, hospitalization rate, severity of COVID-19, and certain laboratory parameters are associated with mortality in hemodialysis patients, emphasizing the need for careful monitoring and management of these factors in clinical practice.

### 3.3. Logistic Regression Analysis of Mortality Factors

This study analyzed factors influencing mortality in dialysis patients using logistic regression. Both univariate and multivariate analyses were conducted, focusing on variables with a *p*-value below 0.05 in the multivariate model. The analysis provided odds ratios (ORs), 95% confidence intervals (95% CIs), and *p*-values for each variable. In the univariate analysis, age and dialysis months showed significant effects, with ORs of 1.09 (95% CI: 1.06–1.11, *p* < 0.001) and 1.01 (95% CI: 1.01–1.01, *p* = 0.037), respectively. These factors remained significant in the multivariate analysis, with age having an OR of 1.07 (95% CI: 1.04–1.10, *p* < 0.001) and dialysis months an OR of 1.01 (95% CI: 1.01–1.01, *p* = 0.041). Vaccination status, PHOS, AST, Hb, UA, and F/T showed significance in the univariate analysis but mostly lost significance in the multivariate analysis. For example, vaccination status had an OR of 0.39 (95% CI: 0.15–0.99, *p* = 0.049) in the univariate analysis, but in the multivariate analysis, the OR was 0.56 (95% CI: 0.17–1.17, *p* = 0.355), indicating a weakening effect on mortality when considering other variables (see Table 3).

### 3.4. Mortality Trends

**Figure 2:**
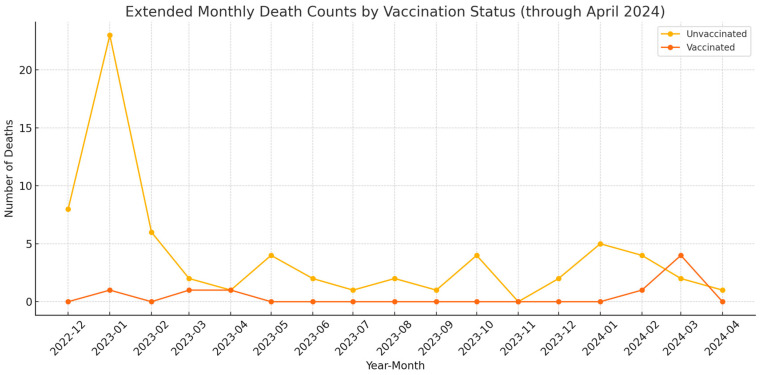
Mortality and vaccination status of hemodialysis patients from December 2022 to April 2024.

According to the data, in December 2022, there were eight deaths among hemodialysis patients, all of whom were unvaccinated. This indicates a significant increase in mortality rates among these patients at the onset of the COVID-19 pandemic. In January 2023, the number of deaths peaked at 24, with 23 unvaccinated and one vaccinated individual, showing the most severe impact of the pandemic during this month. After January 2023, the death counts for both vaccinated and unvaccinated individuals remained generally low and relatively stable, with only occasional minor peaks. Overall, the vast majority of deaths were among unvaccinated individuals, suggesting that vaccination may provide some degree of protection for hemodialysis patients. However, further data and analysis are required to confirm this observation. In summary, December 2022 and January 2023 were the peak months for mortality among hemodialysis patients, followed by a subsequent decline and stabilization in the following months. The higher proportion of deaths among unvaccinated patients suggests that not being vaccinated may increase the risk of death. For detailed results, see Figure 2.

### 3.5. Impact of Vaccination on Survival Rate

**Figure 3:**
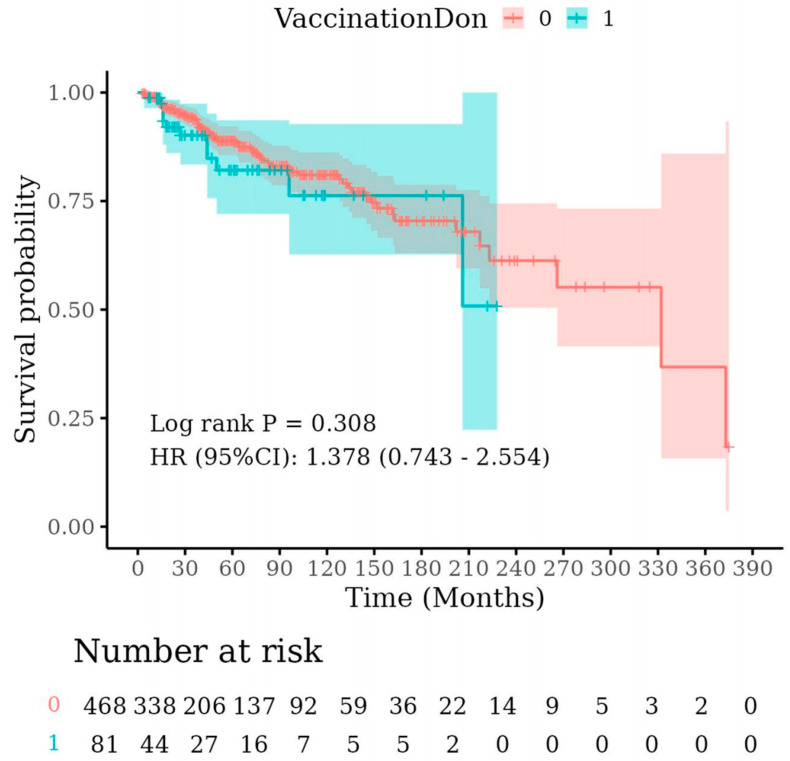
Curves: Blue: Vaccinated group (VaccinationDon = 1), Red: Not Vaccinated group (VaccinationDon = 0); Shaded areas: 95% confidence intervals; log-rank *p* value: 0.308; hazard ratio (HR): 1.378 (95% CI: 0.743–2.554); Number at risk: Indicates the number of patients at risk at each time point.

We analyzed the impact of vaccination (VaccinationDon = 1) on the survival rate of hemodialysis patients; for detailed results, see Figure 3. The vertical axis represents survival probability, and the horizontal axis represents follow-up time (months). The two curves in the figure depict the survival rates of the vaccinated (blue) and unvaccinated (red) cohorts, with shaded areas indicating the 95% confidence intervals. Survival analysis results indicate no statistically significant difference in survival rates between vaccinated and unvaccinated patients (log-rank *p* = 0.308). The hazard ratio (HR) was 1.378, with a 95% confidence interval ranging from 0.743 to 2.554, which encompasses 1, further corroborating the absence of a significant difference in survival rates between the two groups. The “Number at risk” table at the bottom of the figure displays the number of patients at risk at each time point.

An analysis based on the dialysis survival years of hemodialysis patients reveals that the survival curves of the two groups do not differ significantly throughout the follow-up period. Although the survival rate of the vaccinated group was slightly lower than that of the unvaccinated group, statistical analysis indicates that this difference was not significant. In summary, vaccination does not have a statistically significant impact on the long-term survival rate of hemodialysis patients.

### 3.6. Cox Proportional Hazards Regression Analysis

**Figure 4:**
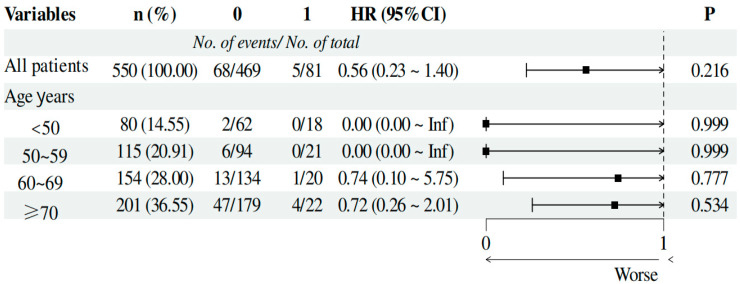
The impact of vaccination on mortality across different age groups. 0: Unvaccinated; 1: Vaccinated; No. of events/No. of total: Number of events/Total number; HR (95% CI): hazard ratio (95% confidence interval). Note: HR: hazard ratio; CI: confidence interval; BNP: B-type natriuretic peptide; AST: aspartate aminotransferase; Cr: creatinine; HsCRP: high-sensitivity C-reactive protein; Kt/V: dialysis urea clearance index; ALB: albumin; Ca: calcium; PHOS: phosphate; UA: uric acid; F/M: female/male ratio.

This study employed univariate and multivariate Cox proportional hazards regression analyses to evaluate the impact of several clinical variables on the survival rates of hemodialysis patients. The univariate analysis results indicated that age, BNP, AST, urea, Cr, HsCRP, Kt/V, ALB, Ca, PHOS, ferritin, UA, and the female/male (F/M) ratio significantly affected patient survival (see Table 4). However, in the multivariate analysis, only age remained significant, indicating that age is the only independent predictor (see Table 4). Further analysis of the impact of vaccination on mortality risk, grouped by age, suggests that vaccination may reduce the risk of death across different age groups. However, due to the small sample size in the vaccinated groups, the results lack statistical significance. This indicates the potential benefits of vaccination. For more details, see Figure 4.

### 3.7. Covariance Analysis of Clinical Indicators

A covariance analysis was conducted to evaluate the differences in various clinical indicators between dialysis patients who received the COVID-19 vaccine and those who did not, comparing data from 2022 and 2024. The results indicated statistically significant differences in the changes in LDL and Cr levels between the vaccinated and unvaccinated groups over the two-year period. Specifically, the adjusted mean difference for LDL was 0.18 (95% CI: 0.021 to 0.34, *p* = 0.027), and for Cr, the adjusted mean difference was −87.41 (95% CI: −143.50 to −31.32, *p* = 0.002). Other clinical indicators did not show statistically significant differences between the groups. See Table 5 for details.

## 4. Discussion

Many countries have developed more effective vaccination strategies for hemodialysis patients, including the use of mRNA vaccines with higher immunogenicity [18] and booster doses [19]. In China, however, the majority of hemodialysis patients have only received the traditional inactivated virus vaccine, i.e., CoronaVac. Furthermore, the efficacy of existing vaccines has been compromised by the emergence of new variants of the virus [20]. In the current context dominated by the Omicron variant, it remains uncertain whether CoronaVac can provide long-term protection for the hemodialysis population. More data are needed to confirm the clinical efficacy of CoronaVac in the hemodialysis population.

CoronaVac, one of the earliest vaccines produced in China, was approved by the World Health Organization on June 1, 2021, as one of the initial vaccines against COVID-19. During the early stages of the pandemic, CoronaVac was proven to be effective [21]. It was widely accepted in many countries and regions due to its good safety profile, tolerability, cost-effectiveness, and low incidence of adverse events [22]. As the virus mutated, its immunological efficacy waned [20,23,24]. Additionally, clinical observations have shown that COVID-19 significantly impacts the renal prognosis of hemodialysis patients, prompting us to conduct this retrospective cohort study. Among older adults, the seropositivity rate three months after the second dose was 100% for BNT162b2, 90% for ChAdOx1, and 60% for CoronaVac [25]. Our study also found no statistically significant impact of CoronaVac vaccination on the survival time of hemodialysis patients, consistent with the results mentioned above (Figure 2). Further studies have evaluated the immunological effects of different doses of CoronaVac. Phoom Narongkiatikhun et al. found that two doses of CoronaVac improved the seroconversion rate of anti-spike RBD IgG antibodies in hemodialysis patients [26]. Another study in Singapore showed that three doses of inactivated SARS-CoV-2 vaccine provided greater protection than two doses but offered less protection than three doses of mRNA vaccine [27]. Our research indicated no statistically significant impact of the number of CoronaVac doses on the survival time of hemodialysis patients (Table 2). Differences in patient ethnicity might be a crucial factor contributing to the inconsistent results [28]. Additionally, Phoom Narongkiatikhun et al.’s study involved a maximum of two doses and did not follow up on patients’ long-term survival outcomes. The Singapore study population was not composed of hemodialysis patients. Our study specifically focused on vaccine administration and survival outcomes in hemodialysis patients but did not measure and compare patients’ serum antibody levels, making it difficult to accurately assess vaccine efficacy based solely on outcome events. Therefore, it is necessary to include more parameters to further evaluate the efficacy of inactivated vaccines in hemodialysis patients.

First, the mortality rate in the vaccinated group was significantly lower, further confirming the protective effect of the vaccine [12,13,29]. However, the vaccinated group was significantly younger than the unvaccinated group. This age difference should be considered when interpreting the results. Age is a significant independent predictor of mortality, consistent with many studies indicating that older populations are at higher risk of severe outcomes from COVID-19 (Table 1, Table 2, Table 3 and Table 4). We further analyzed the impact of vaccination on mortality risk by age groups (under 50, 50–60, 60–70, and over 70) and found that vaccination appears to reduce the risk of death across different age groups. However, due to the small sample size of the vaccinated groups, the results did not reach statistical significance (Figure 4). Therefore, the protective effect of the vaccine against COVID-19 infection still requires further investigation. Second, most laboratory indicators showed negative impacts post-vaccination, but the increase in uric acid levels in vaccinated patients was higher than in unvaccinated patients (Table 1), suggesting that uric acid may play a role in the immune response following vaccination. However, this finding requires further investigation to determine any causal relationship. Finally, there was a statistically significant difference in LDL and Cr levels between vaccinated and unvaccinated patients (Table 5). These indicators could potentially serve as factors in assessing the effectiveness of vaccination in hemodialysis patients.

In this study, we analyzed the clinical variables affecting the survival rates of 550 hemodialysis patients. Compared with survivors, deceased patients had significantly lower levels of Hb, ALB, PHOS, and PTH (*p* < 0.05) (Table 2). Previous studies have reported that the incidence of hypophosphatemia is particularly significant in end-stage renal disease patients infected with SARS-CoV-2, and it may be associated with increased mortality in severe cases [21,30]. The imbalance of phosphate levels in hemodialysis patients can be attributed to various factors, including insufficient nutritional intake, impaired nutrient absorption, respiratory alkalosis, vitamin D deficiency, and obesity [31]. SARS-CoV-2 may disrupt the balance of phosphate, calcium, and parathyroid hormone and has been identified as a potential cause of hypoparathyroidism [32]. Long-term follow-up data (Table 5) revealed significant differences in LDL-C and Cr between the vaccinated and unvaccinated groups. Previous studies have shown that SARS-CoV infection reduces the expression of ACE2, a mechanism similar to that of SARS-CoV-2 [33]. Sergio Triana et al. found that SARS-CoV-2 infection downregulates ACE2 expression in the gut, leading to reduced angiotensin II levels, which in turn suppresses PCSK9 activity on LDL receptors, resulting in elevated LDL-C levels [34,35]. Research by Valéria O Silva et al. demonstrated that two doses of CoronaVac inhibit the binding of RBD to ACE2, blocking the aforementioned pathway and reducing the impact on LDL levels [36]. This suggests that COVID-19 vaccination may have a positive effect on lipid metabolism, helping to lower cardiovascular risk. Currently, it has been found that patients infected with COVID-19 experience an increase in blood Cr levels [37]. However, the selected patients are all long-term dialysis patients, with an average Cr value of 8.36 (6.36, 10.44) mg/dl, which is 6 to 10 times higher than that of healthy individuals. For healthy adults, the normal creatinine levels are typically about 0.74 to 1.35 mg/dL for men and 0.59 to 1.04 mg/dL for women. Therefore, the levels of Cr are more closely related to nutritional indicators such as P and ALB. The increase in Cr observed in vaccinated patients may be linked to improvements in their nutritional status. Literature reports indicate that nutritional status deteriorates significantly in dialysis patients post-COVID-19 infection, with notable declines in albumin and creatinine levels. Vaccination may help improve these parameters [38].

We believe that there are three main reasons for these results. First, the dialysis population is inherently immunosuppressed [6]. Factors such as the uremic environment, aging, oxidative stress, intestinal permeability, and deficiencies in vitamin D and EPO all contribute to altered immunity in these patients. Successful vaccination depends on the vaccine’s ability to induce antibodies and cytotoxic T cells. However, these factors interfere with antigen-specific immune responses, including the activation and proliferation of antigen-presenting cells like dendritic cells and/or macrophages, as well as T and B cells, potentially affecting vaccine efficacy [39]. Additionally, it takes longer for hemodialysis patients to reach peak antibody titers post-vaccination compared with the general population [40], and their serum antibody titers are lower [40]. Furthermore, hemodialysis patients experience an earlier and higher rate of antibody titer decline against SARS-CoV-2 than the general population [41]. These factors result in reduced vaccine effectiveness in the dialysis population compared with the general population. Second, the emergence of COVID-19 variants has further diminished the efficacy of existing vaccines. The effectiveness of inactivated virus vaccines against the Omicron variant is significantly lower compared with the Delta variant. Third, the reluctance of the dialysis population to adhere to the recommended number of vaccine doses may also contribute to reduced vaccine efficacy [42,43]. Concerns about vaccine side effects and doubts about their effectiveness are the primary reasons for hesitancy among dialysis patients.

Compared with previous studies, our research assesses the long-term protection offered by CoronaVac to patients undergoing hemodialysis. We also collected various clinical and demographic parameters of the study participants, which may aid in developing COVID-19 prevention and control strategies for hemodialysis patients. However, our study has certain limitations. Firstly, the patient sample was derived from a single institution, and all participants were Chinese, with a limited number of vaccinated participants. This limitation reduces the statistical power to analyze differences between groups. Secondly, our study is retrospective, which introduces potential confounding factors, such as age and gender, and there might be information bias from some oral inquiries. This study only investigated CoronaVac and did not examine populations that received mRNA or vector vaccines, which is one of the limitations of this research. Lastly, we were unable to dynamically monitor the patients’ test indicators before and after vaccination, making it difficult to quantitatively assess the impact of the vaccine on the patients’ conditions.

The peak in mortality rates in December 2022 and January 2023 aligns with the known surge in COVID-19 cases, and the subsequent decline may be attributed to increased immunity within the population due to natural infection and vaccination. Most deaths occurred among unvaccinated patients, further emphasizing the critical role of vaccination in mitigating severe outcomes. However, the lack of significant difference in long-term survival rates between vaccinated and unvaccinated groups could be influenced by various factors, including patients’ health status and the efficacy of treatments provided during the pandemic. Age emerged as a significant independent predictor of mortality, consistent with many studies indicating a higher risk of severe outcomes from COVID-19 among the elderly. The vaccinated group was notably younger than the unvaccinated group, contributing to the significantly lower mortality rate observed in the vaccinated cohort. Thus, age must be considered when interpreting these findings. Future research should focus on observing the immunogenicity of CoronaVac in the dialysis population.

## 5. Conclusions

Our study indicates that CoronaVac provides a certain level of long-term protection for hemodialysis patients and that vaccination plays a critical role in mitigating severe outcomes of COVID-19. Despite some limitations, such as the single-source sample, inherent biases of a retrospective study, and limited dynamic monitoring, our findings offer valuable insights for developing COVID-19 prevention and control strategies for hemodialysis patients. Future research should continue to observe the immunogenicity of CoronaVac in the dialysis population to further understand the long-term benefits and protective mechanisms of the vaccine.

## Figures and Tables

**Table 1:** Demographic and clinical characteristics of hemodialysis patients by COVID-19 vaccination status.

Variables	Total (*n* = 550)	Unvaccinated (*n* = 469)	Vaccinated (*n* = 81)	*p*-Value
Gender, *n* (%)				0.209
Male	204 (37.09)	179 (38.17)	25 (30.86)	
Female	346 (62.91)	290 (61.83)	56 (69.14)	
Age, Y (Q_1_, Q_3_)	65.00 (54.25, 75.00)	65.00 (56.00, 75.00)	60.00(51.00, 71.00)	0.005 **
Age Group, *n* (%)				0.054
<50 years	80 (14.55)	62 (13.22)	18 (22.22)	
50~59 years	115 (20.91)	94 (20.04)	21 (25.93)	
60~69 years	154 (28.00)	134 (28.57)	20 (24.69)	
≥70 years	201 (36.55)	179 (38.17)	22 (27.16)	
Infected (%)				0.199
No	133 (28.06)	115 (29.26)	18 (22.22)	
Yes	341 (71.94)	278 (70.74)	63 (77.78)	
Hospitalization, *n* (%)				0.987
No	468(85.25%)	399 (85.26)	69 (85.19)	
Yes	81 (14.75)	69 (14.74)	12 (14.81)	
Death, *n* (%)				0.041 *
No	477 (86.73)	401 (85.50)	76 (93.83)	
Yes	73 (13.27)	68 (14.50)	5 (6.17)	
UA (mg/dL)	6.76 ± 1.75	6.68 ± 1.73	7.29 ± 1.80	0.009 **
Cr (mg/dL)	8.36 (6.36,10.44)	8.36 (6.36,10.36)	8.43 (6.40,10.98)	0.668
BNP (pg/mL)	631.80 (172.00, 1854.90)	706.00 (198.00, 2123.00)	303.50 (134.75, 1318.50)	0.094
KT/V	1.45 ± 0.36	1.45 ± 0.35	1.41 ± 0.38	0.412
LDL-C (mg/dL)	2.19 ± 0.81	2.17 ± 0.80	2.29 ± 0.89	0.287
PTH (pg/mL)	228.70 (117.40, 438.70)	233.40 (121.30, 454.25)	187.00 (98.10, 367.30)	0.059
PHOS (mg/dL)	1.61 ± 0.46	1.59 ± 0.47	1.67 ± 0.42	0.168
PT (seconds)	11.30 ± 1.99	11.34 ± 2.11	11.04 ± 0.86	0.303
Hb (g/dL)	10.97 ± 1.37	10.98 ± 1.37	10.94 ± 1.39	0.818
ALB(g/L)	35.58 ± 4.00	35.48 ± 3.92	36.23 ± 4.43	0.169
TCH (mg/dL)	3.89 ± 1.10	3.89 ± 1.09	3.90 ± 1.17	0.939
AST (U/L)	18.94 ± 8.78	19.10 ± 8.91	17.97 ± 7.91	0.351
Ca (mg/dL)	2.22 ± 0.20	2.23 ± 0.20	2.18 ± 0.17	0.082
Na (mmol/L)	138.07 ± 3.22	138.02 ± 3.18	138.37 ± 3.50	0.366
β2-MG (mg/L)	32.92 ± 8.09	33.16 ± 7.97	31.30 ± 8.78	0.164
Ferritin (ng/mL)	80.75 (39.08, 161.20)	76.05 (38.08, 160.82)	106.20 (55.52, 171.18)	0.090

Values are mean ± SD, M (Q_1_, Q_3_) or number (%), *p* < 0.05 was deemed significant. (SD: standard deviation, M: Median, Q_1_: 1st Quartile, Q_3_: 3st Quartile), * *p* < 0.05, ** *p* < 0.01. UA, uric acid; Cr, creatinine, B-type natriuretic peptide; KT/V, urea clearance index; LDL-C, low-density lipoprotein cholesterol; PTH, parathyroid hormone; PHOS, phosphate; PT, prothrombin time; Hb, hemoglobin; TCH, total cholesterol; AST, Aspartate Aminotransferase; Ca, calcium; Na, natrium; β2-MG, β 2-Microglobulin.

**Table 2:** Demographic and clinical characteristics of hemodialysis patients by death status.

Variables	Total (*n* = 550)	Survivor Group (*n* = 477)	Deceased Group (*n* = 73)	*p*-Value
Gender, *n* (%)				0.810
Male	204 (37.09)	176 (36.90)	28 (38.36)	
Female	346 (62.91)	301 (63.10)	45 (61.64)	
Age, M (Q_1_, Q_3_)	65.00 (54.25, 75.00)	63.00 (53.00, 72.00)	77.00 (67.00, 87.00)	<0.001 **
Age Group, *n* (%)				<0.001 **
<50 years	80 (14.55)	78 (16.35)	2 (2.74)	
50~59 years	115 (20.91)	109 (22.85)	6 (8.22)	
60~69 years	154 (28.00)	140 (29.35)	14 (19.18)	
≥70 years	201 (36.55)	150 (31.45)	51 (69.86)	
Hospitalization, *n* (%)				<0.001 **
No	468 (85.25)	417 (87.42)	51 (70.83)	
Yes	81 (14.75)	60 (12.58)	21 (29.17)	
Severe COVID-19 and Death, *n* (%)				<0.001 **
No	530 (96.36)	472 (98.95)	58 (79.45)	
Yes	20 (3.64)	5 (1.05)	15 (20.55)	
Vaccination Don, *n* (%)				0.041 *
No	469 (85.27)	401 (84.07)	68 (93.15)	
Yes	81 (14.73)	76 (15.93)	5 (6.85)	
Vaccine Number, *n* (%)				0.264
0	469 (85.27)	401 (84.07)	68 (93.15)	
1	23 (4.18)	21 (4.40)	2 (2.74)	
2	28 (5.09)	26 (5.45)	2 (2.74)	
3	30 (5.45)	29 (6.08)	1 (1.37)	
ALB(g/L)	35.58 ± 4.00	35.94 ± 3.85	33.44 ± 4.19	<0.001 **
UA (mg/dL)	4.55 ± 1.18	4.65 ± 1.12	3.93 ± 1.31	<0.001 **
Cr (mg/dL)	8.36 (6.36, 10.44)	8.80 (6.63, 10.83)	7.16 (5.59, 8.70)	<0.001 **
BNP (pg/mL)	631.80 (172.00, 1854.90)	452.50 (150.07, 1730.00)	989.00 (495.25, 2408.40)	0.009 **
KT/V	1.45 ± 0.36	1.46 ± 0.36	1.36 ± 0.29	0.123
LDL-C (mg/dL)	2.19 ± 0.81	2.21 ± 0.83	2.04 ± 0.68	0.115
PTH (pg/mL)	228.70 (117.40, 438.70)	224.80 (117.43, 416.90)	293.80 (114.15, 511.45)	0.307
PHOS (mg/dL)	1.61 ± 0.46	1.64 ± 0.46	1.41 ± 0.46	<0.001 **
PT (seconds)	11.30 ± 1.99	11.20 ± 2.03	11.87 ± 1.65	0.015 *
Hb (g/dL)	10.97 ± 1.37	11.06 ± 1.33	10.44 ± 1.51	<0.001 **
TCH (mg/dL)	3.89 ± 1.10	3.93 ± 1.12	3.64 ± 0.98	0.050
AST (U/L)	18.94 ± 8.78	18.33 ± 7.74	22.42 ± 12.76	0.014 *
Ca (mg/dL)	2.22 ± 0.20	2.22 ± 0.20	2.20 ± 0.17	0.280
Na (mg/L)	138.07 ± 3.22	138.15 ± 3.26	137.50 ± 2.93	0.111
β2-MG (mg/L)	32.92 ± 8.09	32.84 ± 7.93	33.55 ± 9.28	0.612
Ferritin (ng/mL)	132.12 ± 170.58	121.21 ± 139.19	198.16 ± 290.22	0.058

Values are mean ± SD, M (Q_1_, Q_3_) or number (%), *p* < 0.05 was deemed significant (SD: standard deviation, M: Median, Q_1_: 1st Quartile, Q_3_: 3st Quartile), * *p* < 0.05, ** *p* < 0.01.

**Table 3:** Factors influencing mortality in dialysis patients: logistic regression analysis.

Variables	Univariate Analysis				Multivariate Analysis			
	OR	95%CI		*p*-Value	OR	95%CI		*p*-Value
Age	1.09	1.06	1.11	<0.001 **	1.07	1.04	1.10	<0.001 **
Dialysis Months	1.01	1.01	1.01	0.037 *	1.01	1.01	1.01	0.041 *
Vaccination Don								
No	1.00				1.00			
Yes	0.39	0.15	0.99	0.049 *	0.56	0.17	0.17	0.355
PHOS (mg/dL)	0.28	0.15	0.53	<0.001 **	0.99	0.42	2.29	0.972
AST (U/L)	1.04	1.02	1.07	<0.001 **	1.03	0.99	1.07	0.158
Hb (g/dL)	0.97	0.95	0.95	<0.001 **	0.98	0.95	1.00	0.091
UA (mg/dL)	0.99	0.99	0.99	<0.001 **	1.00	0.99	1.00	0.102
F/T	0.97	0.95	0.99	0.027 *	0.98	0.95	1.00	0.075

Variables considered predictors of mortality were those with a *p*-value below 0.05 in the multivariate model. The table details odds ratios (ORs), their 95% confidence intervals (95% CIs), and *p*-values. Note: “Not Vaccinated” serves as the control group. CI: confidence interval, OR: odds ratio.* *p* < 0.05, ** *p* < 0.01.

**Table 4:** Cox proportional hazards regression analysis of clinical variables on survival rates of hemodialysis patients.

Variable	Univariate Analysis				Multivariate Analysis			
	HR	95%CI		*p*-Value	HR	95%CI		*p*-Value
Age	1.09	1.07	1.11	<0.001 **	1.31	1.01	1.71	0.048 *
BNP (pg/mL)	1.01	1.01	1.01	0.028 *	1.00	1.00	1.00	0.070
AST (U/L)	1.06	1.04	1.08	<0.001 **	1.09	0.84	1.42	0.509
Urea (mg/dL)	0.94	0.90	0.98	0.004 **	0.82	0.51	1.33	0.422
Cr (mg/dL)	0.99	0.99	0.99	<0.001 **	1.00	0.99	1.01	0.610
Hscrp (mg/L)	1.02	1.01	1.02	<0.001 **	0.87	0.62	1.22	0.423
KT/V	0.38	0.22	0.67	<0.001 **	0.02	0.00	67.52	0.344
ALB (g/dL)	0.85	0.80	0.89	<0.001 **	1.01	0.63	1.62	0.966
Ca (mg/dL)	0.19	0.05	0.64	0.008 **	0.63	0.00	101.01	0.857
PHOS (mg/dL)	0.20	0.11	0.37	<0.001 **	1.66	0.06	49.60	0.770
Ferritin (ng/mL)	1.01	1.01	1.01	0.003 **	1.00	0.99	1.02	0.485
UA (mg/dL)	0.99	0.99	0.99	<0.001 **	1.01	0.98	1.03	0.508
F/M	0.97	0.95	0.99	0.047 *	0.84	0.68	1.04	0.102

* *p* < 0.05, ** *p* < 0.01.

**Table 5:** Covariance analysis of clinical indicators in vaccinated and unvaccinated dialysis patients (2022–2024).

Variable	Unvaccinated		Vaccinated		LS Mean Difference (95%CI)	*p*-Value
	No.	Change from 2022, Mean Difference (SD)	No.	Change from 2022, Mean Difference (SD)		
ALB	342	1.28 (3.86)	60	1.65 (4.66)	−0.593 (−1.52–0.33)	0.209
ALT	338	−1.46 (10.03)	60	0.28 (24.35)	−2.105 (−5.22–1.01	0.185
Urea	345	−0.23 (8.18)	62	−1.60 (7.01))	1.10 (−0.72–2.93)	0.236
LDL-C	320	−0.084 (0.59)	56	−0.34 (0.90)	0.18 (0.021–0.34)	0.027 *
TG	335	−0.027 (1.50)	59	−0.13 (1.22)	0.136 (−0.24–0.51)	0.474
HDL-C	335	−0.085 (0.20)	59	−0.098 (0.21)	0.018 (−0.031–0.067)	0.473
Cr	345	46.50 (220.73)	62	129.55 (270.40))	−87.41 (−143.50–−31.32)	0.002 **
PTH	369	−33.57 (259.86)	66	1.89 (194.16)	5.37 (−46.54–57.27)	0.839
PHOS	400	0.108 (0.50)	71	0.105 (0.52)	−0.034 (−0.14–0.071)	0.523
PT	307	0.55 (6.77)	50	0.48 (1.70)	0.33 (−1.49–2.16)	0.721
PA	281	−15.56 (68.24)	44	−12.43 (71.10)	−7.074 (−27.25–13.10)	0.491
AST	329	6.67 (111.05)	59	2.24 (38.36)	4.77 (−23.99–33.53)	0.744
Ferritin	297	−15.16 (199.77)	49	−42.28 (126.10)	10.70 (−33.65–55.05)	0.635
β2-MG	244	1.05 (8.33)	38	1.23 (7.97)	0.341 (−2.28–2.91)	0.812
Hb	412	−0.70 (14.91)	77	0.86 (15.41)	−1.10 (−3.93–1.73)	0.446
UA	341	−4.45 (111.71)	63	−11.24 (135.38)	−11.31 (−38.64–16.02)	0.416
TCH	335	−0.26 (0.93)	58	−0.41 (1.14)	0.15 (−0.075–0.38)	0.189
BNP	35	572.90 (1555.47)	7	223.32 (896.66)	549.73 (−664.84–1764.30)	0.366

* *p* < 0.05, ** *p* < 0.01.

## Data Availability

The data supporting the reported results can be found.

## Supplementary Materials

The following supporting information can be downloaded at https://www.mdpi.com/article/10.3390/vaccines12070799/s1, Table S1: Fundamental Data on Vaccines and Dialysis (550).
